# The effect of non-verbal music on anxiety in hospitalized children

**DOI:** 10.1186/s12887-023-04101-2

**Published:** 2023-06-05

**Authors:** Ashrafalsadat Hakim, Seyedeh Shima Hosseini Kaldozkhi, Ashraf Tashakori, Saeed Ghanbari

**Affiliations:** 1 Nursing Care Research Center in Chronic Diseases, Department of Nursing, Ahvaz Jundishapur University of Medical Sciences, Ahvaz, Iran; 2 Master of Nursing Student, School of Nursing and Midwifery, Ahvaz Jundishapur University of Medical Sciences, Ahvaz, Iran; 3 Department of Psychiatry, School of Medicine, Ahvaz Jundishapur University of Medical Sciences, Ahvaz, Iran; 4 Department of Health and Epidemiology, School of Public Health, Ahvaz Jundishapur University of Medical Sciences, Ahvaz, Iran

**Keywords:** Non-verbal music, Anxiety, Vital signs, Hospitalized children

## Abstract

**Background:**

In recent years, the positive effect of non-pharmacological methods such as listening to music in reducing the level of anxiety of hospitalized patients has been reported. This study aimed to determine the effect of non-verbal music on anxiety in hospitalized children.

**Methods:**

In this study, 52 hospitalized children aged 6 to 12 years were randomly divided into Test and control groups. Research data collection tools included the Spielberger questionnaire to assess the level of anxiety in children. Statistical analysis of data was performed using Chi-square and t-tests by SPSS 23 software.

**Results:**

Daily listening to non-verbal music for 20 minutes after the second and third days significantly reduced the anxiety score and the number of breaths per minute of hospitalized children (P ≤ 0.01). The trend of changes in anxiety score was measured for three consecutive days and vital signs except body temperature decreased significantly in the test group (P ≤ 0.01).

**Conclusion:**

According to the results of this study, listening to non-verbal music by hospitalized children can be used as an effective practical method to reduce the level of anxiety and subsequently reduce vital signs.

## Introduction

According to the World Health Organization reports, more than 6 million children are hospitalized each year for a variety of reasons [1]. In general, every child is hospitalized at least once in their lifetime due to illness [2]. So in addition to the importance of hospitalizing the child to achieve their health, the hospital environment for various reasons such as the color and smell of the environment, hospital staff coverage, separation from friends and caregivers, etc. can be emotionally and mentally unpleasant for them[3]. This problem doubles in children between 6 and 12 years old due to more mental, emotional, and social development [4]. Stress and anxiety increase the secretion of epinephrine hormones by stimulating the sympathetic nerves and ultimately altering vital signs including heart rate, blood pressure, respiration rate, and body temperature [5, 6].

Anxiety and subsequent changes in physiological responses in children can cause side effects such as prolonged recovery time and the need to take sedatives [7]. The anxiety caused by children’s hospitalization causes damage to their biological and psychological development [2]. Therefore, due to this issue, in recent years, pharmacological and non-pharmacological methods have been proposed for the mental adaptation of children to the hospital environment [8, 9]. Among non-pharmacological methods, music therapy is a non-invasive treatment method provided to patients by nurses [10]. Music therapy can reduce anxiety and physiological responses in hospitalized patients (1, 2, 9, and 11). Music affects the brain and stimulates alpha waves, releasing endorphins and ultimately reducing anxiety and relieving pain [9].

The results of studies performed on hospitalized children show that music significantly reduces their anxiety (12, 13, and 14). Jordanovo et al. reported that music therapy could be used as an intervention to reduce preoperative anxiety in children with leukemia [15]. In their study, Roslita and colleagues found that listening to music improved the physiological responses in children with asthma who received inhalation therapy [16]. Despite the effect and role of music in reducing anxiety in hospitalized children, limited studies have been conducted in this field. Also, in previous studies, the effect of music on the anxiety of hospitalized children has been studied only one time. While in the present study, music intervention was performed for three consecutive days to check the manner of changing vital signs over time. Therefore, in this study, the effect of non-verbal music on anxiety in hospitalized children was evaluated.

## Methods

The present study is an experimental study. A total of 52 children aged 6 to 12 years admitted to educational hospitals (pediatric general ward) in 2022 were randomly divided into Test (n = 26) and control (n = 26) groups. Inclusion criteria included (1) children aged 6 to 12 years, and (2) Children who were hospitalized in the general pediatric ward. Exclusion criteria included: (1) children with hearing problems, (2) children with mental health problems who needed sedation, and (3) children admitted to the intensive care unit who wanted to undergo surgery. Before the intervention, by receiving the code of ethics (IR.AJUMS.REC.1399.078) from the Vice Chancellor for Research, the details of 52 children with inclusion criteria were received from one of the educational hospitals. It should be noted that all the methods were performed according to the relevant guidelines and regulations. Also, before starting the study, the objectives of the study were explained to the children and their parents. Then informed consent was obtained from all the parents of the children to start the research. Demographic information of children that included age and gender was recorded with the help of their parents. Johann Sebastian’s non-verbal music was provided to children admitted to the pediatric general ward, and with the help of nurses and parents, children were asked to spend 20 minutes every day for three days between 2 pm and 3 pm (due to the absence of visitors) Listening to it using an MP3 player and headphones.

Johann Sebastian’s non-verbal music consists of different parts and in this research, episode three of this work was used due to its smooth and flat movement. Simultaneously with listening to music for 20 minutes in the test group, headphones without music to avoid disrupting the intervention process were placed in the ears of children in the control group for 20 minutes. Children were asked to lie on their beds in any position they felt most comfortable (sitting or lying down) and to listen to the music with their eyes closed. During these three days, the researcher evaluated and recorded the vital signs of children in both groups every day before and after the intervention. Using the Spielberger questionnaire [17], the level of anxiety of children in the two groups was assessed. Questionnaires and vital signs were completed by the researcher before the intervention and every day for three days. Meanwhile, vital signs were checked immediately before the intervention, and then 0.5 hours after the music, vital signs were checked. All participants in the control group received routine ward care and put headphones on their ears without an earphone. Also, the vital signs of the control group were taken. It should be noted that to observe ethical considerations, after conducting music therapy sessions and filling out questionnaires, a music therapy piece was given to the control group.

Research data collection tools included the Spielberger questionnaire [17] to assess the level of anxiety in children, demographic information registration questionnaire (age and sex), and patient vital signs registration form (systolic blood pressure, diastolic blood pressure, body temperature, Heart rate, and respiration). The Spielberger Questionnaire consists of 40 questions in two modes and attributes (20 questions each) that assess the level of anxiety. In this study, only the section on measuring state anxiety was used. The maximum and minimum scores for each state anxiety test were 60 and 20 points, respectively. The child was asked to express his/her feelings on three scales: very high (3 points), medium (2 points), and rarely (1 point). Scores less than or equal to 33 indicate mild and equal anxiety, scores greater than 47 indicate severe anxiety, and scores between 32 and 46 indicate moderate anxiety. Vital signs including heart rate, systolic blood pressure, diastolic blood pressure, respiration rate, and body temperature were monitored and recorded on a hospital bed monitor.

To confirm the validity of blood pressure recorded by the monitor, a calibrated mercury sphygmomanometer (made in Germany) was used for all patients in the study. Pulse and number of breaths were counted using the hour counter in full minutes. The child’s body temperature was measured by a sublingual thermometer. To assess the reliability of vital signs, after opening the cuff of the sphygmomanometer and for five minutes without changing the location of the cuff, the vital signs were checked again by another nurse colleague and recorded in the datasheet. The reliability coefficient of simultaneous observation was calculated to be about 95%. Quantitative variables were reported as mean and standard deviation and qualitative variables were reported as numbers (percentage). A Chi-square test was used to examine the relationship between qualitative variables and a t-test was used to compare the mean of quantitative variables between the two groups. The process of changes in variables was examined using a repeated-measure analysis of variance. Statistical analysis of data was performed by SPSS_23_ software. The significance level of the above tests was considered less than 0.05. The process of changes in variables was examined using a repeated-measures analysis of variance.

## Results

As shown in Table 1, out of 52 children participating in this study, 27 (51.9%) were boys and 25 (48.1%) were girls. According to the Chi-square test, no significant difference was observed between the control and intervention groups in terms of their mean age. Also, based on the test, it was found that there is no statistically significant difference between the two groups of control and intervention in terms of gender (Table 1). The changes in the mean score of anxiety in the intervention group decreased (about 12 points on the third day compared to the first day) and were statistically significant (P = 0.000), while in the control group were not significant (P = 0.631) (Table  2).

The changes in mean systolic blood pressure measured in the control group were not significant (P = 0.555), but in the test group, the trend of changes in systolic blood pressure was significantly reduced and affected by music (P = 0.000). Based on statistical analysis by repeated-measures analysis of variance, heart rate changes during three consecutive days were not affected in the control group (P = 0.756), while in the test group, the heart was significantly decreased for three consecutive days under the influence of music (P = 0.001). The results show that the number of breaths in both groups before the intervention was equal (P = 0.765). this parameter decreased at other times including the first day after the intervention and the second and third days before and after the intervention in children hospitalized compared to the control group (Table  3). Also, based on the results of repeated measures analysis of variance, the mean number of breaths in the intervention group decreased (P = 0.101), but not in the control group (p-0.000).

The results of the statistical analysis of body temperature show that the difference between the mean body temperature between the test and control groups at any time before and after the intervention and every three days was not significant (Table 3). Also, changes in the mean temperature of the body were examined at different times and were not statistically significant in both groups (P = 0.153 and P = 0.246, respectively).

**Table 1:** Demographic characteristics of hospitalized children participating in the study

Gender, number (%)	group		p-value^1^
	Control	Test	
Female	14 (53.8)	11(42.3)	0.0405
male	12 (46.2)	15 (57.3)	
Age (year), mean ± standard deviation	9.3 ± 1.85	8.9 ± 2.12	0.384

Chi-square and t-tests were used to analyze gender and age variables, respectively

**Table 2:** The effect of non-verbal music on the level of anxiety of hospitalized children

Anxiety level, mean ± standard deviation	Group		p-value
	Control	Test	
First day-before intervention	45.1 ± 7.77	46.0 ± 6.35	0.655
First day-after intervention	44.9 ± 6.29	42.1 ± 5.4	0.095
s day-before intervention	44.3 ± 6.65	41.9 ± 6.00	0.188
s day-after intervention	43.9 ± 7.00	38.1 ± 4.98	0.001
Third day-before intervention	42.4 ± 5.96	37.5 ± 5.54	0.003
Third day-after intervention	42.1 ± 6.29	34.4 ± 3.40	0.000
RM-ANOVA^2^	0.631	0.000	

1 t-test was used

2 Analysis of variance with repeated measures

**Table 3:** The effect of non-verbal music on vital signs of hospitalized children

Systolic blood pressure, mean ± standard deviation	Group		p-value^1^
	Control	Test	
First day-before intervention	104.5 ± 9.67	103.9 ± 10.00	0.823
First day-after intervention	103.0 ± 11.4	101.2 ± 10.42	0.547
s day-before intervention	104.1 ± 12.29	100.1 ± 8.60	0.176
s day-after intervention	103.6 ± 11.63	97.3 ± 10.60	0.046
Third day-before intervention	99.7 ± 10.46	97.0 ± 9.25	0.324
Third day-after intervention	98.9 ± 10.1	92.9 ± 8.48	0.025
RM-ANOVA	0.631	0.000	
Diastolic blood pressure, mean ± standard deviation	Control	Test	P-Value
First day-before intervention	68.1 ± 7.57	69.6 ± 8.92	0.506
First day-after intervention	67.9 ± 6.41	61.4 ± 8.07	0.806
s day-before intervention	68.0 ± 5.99	67.1 ± 6.79	0.621
s day-after intervention	67.1 ± 6.00	65.3 ± 5.42	0.240
Third day-before intervention	67.1 ± 6.22	64.9 ± 6.67	0.218
Third day-after intervention	66.6 ± 4.39	63.1 ± 5.05	0.010
RM-ANOVA	0.828	0.009	
**Heart rate, mean ± standard deviation**	Control	Test	P-Value
First day-before intervention	10.8 ± 6.75	10.9 ± 14.51	0.961
First day-after intervention	99.5 ± 7.80	98.1 ± 9.84	0.577
s day-before intervention	99.0 ± 101.52	98.3 ± 9.08	0.801
s day-after intervention	98.3 ± 9.48	94.5 ± 7.03	0.110
Third day-before intervention	97.6 ± 9.58	94.0 ± 6.36	0.115
Third day-after intervention	97.3 ± 10.763	91.1 ± 4.77	0.011
RM-ANOVA	0.765	0.001	
**Respiration rate, mean ± standard deviation**	Control	Test	P-Value
First day-before intervention	24.2 ± 1.63	24.7 ± 3.01	0.469
First day-after intervention	24.8 ± 2.00	22.7 ± 2.28	0.001
s day-before intervention	23.6 ± 1.85	22.1 ± 1.70	0.004
s day-after intervention	23.5 ± 1.50	20.1 ± 2.14	0.000
Third day-before intervention	23.3 ± 1.36	20.3 ± 2.7	0.000
Third day-after intervention	23.2 ± 1.37	19.0 ± 2.04	0.000
RM-ANOVA	0.101	0.001	
Body temperature in degrees Celsius, mean ± standard deviation	Control	Test	P-Value
First day-before intervention	36.7 ± 0.6	36.6 ± 1.02	0.695
First day-after intervention	37.6 ± 0.80	36.4 ± 0.88	0.336
s day-before intervention	36.4 ± 0.93	36.3 ± 1.19	0.730
s day-after intervention	36.5 ± 1.06	36.5 ± 1.61	0.167
Third day-before intervention	36.5 ± 1.08	37.0 ± 0.41	0.047
Third day-after intervention	37.1 ± 0.59	36.6 ± 0.92	0.021
RM-ANOVA	0. 153	0.246	

1 t-test was used

2 Analysis of variance with repeated measures

## Discussion

Fear and anxiety are the main consequences of hospitalizing a child which may have unpleasant and irreparable consequences for the child and the community. The side effects of chemical sedative drugs at the same time with an increase in children’s hospitalization in recent decades have led to encouraging researchers to use non-pharmacological methods, including music therapy, to reduce their anxiety [18–20]. Therefore, in the present study, the aim was to investigate the effect of Johann Sebastian Bach’s non-verbal music on the level of anxiety and vital signs in hospitalized children aged 6–12 years. The results of this study show that from the second day and after the intervention, the level of anxiety in children in the intervention group was significantly lower than in the control group and the trend of changes was decreasing over time.

According to the results, Atak et al. [20], Karbandi et al. [2], Karakol et al. [6] and Rabie et al. [12] reported that the effect of music therapy on children’s anxiety reported that the level of anxiety of hospitalized children in the hospital with the use of music therapy is significantly reduced. In a recent study, Johnson et al. [21] reported that music therapy as an effective and safe non-pharmacological intervention can reduce hospitalized children’s anxiety. Based on these results and in line with the results of the present study, it can be said that Johann Sebastian Bach’s non-verbal music in children in the intervention group has reduced children’s anxiety by providing a relaxing environment. In contrast, in a study by Aydin et al., It was observed that the level of anxiety of hospitalized children was not affected by music therapy [22]. Causes of discrepancies in the results include differences in sample size, measurement tools, and inclusion criteria. Although the exact biological mechanism is not known, it seems that music increases the secretion of endorphins by affecting a specific part of the brain and stimulating alpha waves, which results in reduced pain and anxiety [9, 22].

Music reduces unpleasant feelings in a person by activating several parts of the subcortical areas of the brain, including the dopaminergic system in the middle part of the brain [23]. A direct relationship between anxiety levels and vital signs such as systolic blood pressure, heart rate, and respiratory rate has been reported in various studies [14, 24–26]. The results of the present study showed that before the intervention, the mean score of anxiety and vital signs of hospitalized children were high, but after three days of listening to non-verbal music in line with the level of anxiety, the mean vital signs also decreased significantly.

Found. Consistent with the results of the present study, Da Silva et al. [27] in evaluating the effect of music therapy on the vital signs of hospitalized children, observed changes in systolic and diastolic blood pressure over time were significantly reduced under the influence of listening to non-verbal music. Consistent with the results of the present study, the similarity of the age suffering of the children studied in the present study with the study of Nguyen et al. [28] can confirm the effect of music therapy on reducing heart rate and respiratory rate in children aged 6 to 12 years old. Rajora et al. [29] also observed in their study of adult patients who were mechanically ventilated that music therapy significantly reduced their systolic and diastolic blood pressure. Listening to music by stimulating the parasympathetic nerves reduces the level of vital signs, including blood pressure. [30, 31].

Explaining the results, it can be said that the use of Johann Sebastian Bach’s non-verbal music in children with improved mental and physiological status has led to a significant reduction in systolic and diastolic blood pressure. According to the results of the present study, Nguyen et al. [28] reported that music therapy significantly reduced heart rate and respiration rate per minute in hospitalized children. Decreased heart rate may be related to decreased secretion of catecholamine into the blood as a result of listening to music [32].

## Conclusion

Based on the results and confirming the first hypothesis of the research, listening to Johann Sebastian Bach’s non-verbal music for 20 minutes daily for three consecutive days can significantly reduce the anxiety score of hospitalized children. Also, following the reduction of anxiety due to listening to non-verbal music, the reduction of vital signs including reduction of systolic and diastolic blood pressure, heart rate, and respiration rate per minute was observed which can accelerate the healing process and recovery of children Be hospitalized between 6 and 12 years old.

## Data Availability

The data sets used and/or analyzed during the study are available from the corresponding author.
