# Correction: The effect of non-verbal music on anxiety in hospitalized children

**DOI:** 10.1186/s12887-023-04141-8

**Published:** 2023-06-22

**Authors:** Ashrafalsadat Hakim, Seyedeh Shima Hosseini Kaldozkhi, Ashraf Tashakori, Saeed Ghanbari

**Affiliations:** 1 Nursing Care Research Center in Chronic Diseases, Department of Nursing, Ahvaz Jundishapur University of Medical Sciences, Ahvaz, Iran; 2 Master of Nursing Student, School of Nursing and Midwifery, Ahvaz Jundishapur University of Medical Sciences, Ahvaz, Iran; 3 Department of Psychiatry, School of Medicine, Ahvaz Jundishapur University of Medical Sciences, Ahvaz, Iran; 4 Department of Health and Epidemiology, School of Public Health, Ahvaz Jundishapur University of Medical Sciences, Ahvaz, Iran

**Correction: BMC Pediatr 23, 279 (2023)**

10.1186/s12887-023-04101-2

Following publication of the original article [1], the authors identified errors in the caption of Tables 1 and 2, and in reference citations in page 4 of the published version. The correct Tables 2 and 3 caption, and reference citations are given below.

- Correct Tables 2 and 3 captions:
  - Table 2: The effect of non-verbal music on the level of anxiety of hospitalized children.
  - Table 3: The effect of non-verbal music on vital signs of hospitalized children.
- Correct reference citations:
  - A direct relationship between anxiety levels and vital signs such as systolic blood pressure, heart rate, and respiratory rate has been reported in various studies [14, 24–26].

The original article has been corrected.
